# High prevalence of celiac disease among Saudi children with type 1 diabetes: a prospective cross-sectional study

**DOI:** 10.1186/1471-230X-12-180

**Published:** 2012-12-23

**Authors:** Abdulrahman Al-Hussaini, Nimer Sulaiman, Musa Al-Zahrani, Ahmed Alenizi, Imad El Haj

**Affiliations:** 1 Department of Pediatrics at Children's Hospital, King Fahad Medical City, Riyadh, Kingdom of Saudi Arabia; 2 Children's Hospital, King Saud Medical City, Riyadh, Kingdom of Saudi Arabia; 3 Department of Pathology, King Saud Medical City, Riyadh, Kingdom of Saudi Arabia; 4 University of King Saud for Health Sciences, Division of Pediatric Gastroenterology, Hepatology & Nutrition, Children's Hospital, King Fahad Medical City, PO Box 59046, Riyadh, Postal code 11525, Kingdom of Saudi Arabia

**Keywords:** Celiac disease, Type 1 diabetes, Anti-tissue transglutaminase, Ednomyseal antibody, Saudi Arabia

## Abstract

**Background:**

There is lack of data on prevalence of celiac disease (CD) in children with type 1 diabetes (T1D) in Arabs in the Middle East. The present investigation aims to study the prevalence rate and clinical characteristics of CD among Saudi children with T1D using a combination of the most sensitive and specific screening serologic tests (anti- tissue transglutaminase antibodies IgA [anti-TTG] and ednomyseal antibodies [EMA]) and to determine the lower cut-off value of anti- anti-TTG level that best predicts CD in children with T1D.

**Methods:**

Children with T1D following in diabetic clinic have been prospectively screened for presence of CD, over a two-year period (2008–2010), by doing anti-TTG, EMA, and total IgA. Children with positive anti-TTG titres (>50 U/ml) and/or EMA and children with persistently low positive anti-TTG titres (two readings 20–50 U/ml; within 6 months intervals) had upper endoscopy and 6 duodenal biopsies.

**Results:**

One hundred and six children with T1D have been screened for CD: age ranged between 8 months to 15.5 years (62 females). Nineteen children had positive anti-TTG and/or EMA, however only 12 children had biopsy proven CD (11.3%). Five of 12 had gastrointestinal symptoms (42%). Children with T1D and CD had significantly lower serum iron than children with T1D alone (8.5 μgm/L Vs 12.5 μgm/L; P = 0.014). The sensitivity and specificity of anti-TTG were 91.6% and 93.6%, with a positive and negative predictive value of 64.7% and 98.8%, respectively. Receiver operated characteristics analysis for the best cut-off value of anti-TTG level for diagnosis of CD was 63 units (sensitivity 100% and specificity 98.8%).

**Conclusion:**

CD is highly prevalent among Saudi children with T1D. Anti-TTG titres more than 3 times the upper limit of normal has very high sensitivity and specificity for diagnosis of CD in T1D children.

## Background

Type 1 diabetes mellitus (T1D) and celiac disease (CD) are both autoimmune disorders. In the last two decades, increased CD prevalence (1–16%) in patients with type 1 diabetes mellitus (T1D) has been well documented in numerous screening studies made all over the world [1-5]. There is limited data on the prevalence of CD in Arab children with T1D. Data from Arab countries in North Africa indicate prevalence rate ranging from 5 to 16% [3,6,7]. There is a limited data on prevalence of CD in children with T1D in Arabs in Middle East. Anti-gliadin antibodies-based screening study in Riyadh, the capital city of Saudi Arabia, revealed a 4.9% prevalence of CD in T1D children [8]. Anti-gliadin antibodies-based screening is no longer recommended because of the poor sensitivity and specificity [9]. In a large retrospective, anti-tissue transglutaminase (anti-TTG) antibodies based, screening study, the prevalence of CD among T1D children in Western region of Saudi Arabia was 11.2% [10].

Several studies confirmed that high concentrations of anti-TTG in serum predict villous atrophy better than low values [11-13]. On the other hand, low anti-TTG values have low sensitivity and specificity for diagnosis of CD [14,15]. Children with T1D, like other autoimmune diseases at risk of CD, could have false positive or transiently positive anti-TTG results at low values [16]. Thus children with T1D and low anti-TTG values might be subjected to anesthesia and endoscopy un-necessarily. Therefore, determination of the lower positive cut-off value of anti-TTG that best predict CD in T1D patients might help in proposing an efficient and economical strategy for diagnosing CD.

The two main aims of the present prospective cross-sectional study were to study the prevalence rate and clinical characteristics of CD in Saudi children with T1D using a combination of the most sensitive and specific screening serologic tests (Anti-TTG and EMA), and to determine the lower positive cut-off value of anti-TTG that best predicts histopathological diagnosis of CD in children with T1D.

## Methods

We prospectively enrolled 106 children with T1DM seen in the diabetic clinics of the King Saud Medical City, Riyadh, during the period from June 2008 through January 2010. After explaining the objectives of the study, a written informed consent was obtained from the children’s parents. A physician interviewed parents and performed physical examination for all enrolled patients. During interviews, patients and their parents were asked about persistent gastrointestinal symptoms such as diarrhea, constipation, abdominal distension, vomiting, and abdominal pain over the past year. Gender of the patients, their ages, age at onset of diabetes and duration of diabetes were recorded. Medical chart review focused on results of other antibody tests such as serum antithyroperoxidase, associated diseases, and patient’s age at onset of diabetes.

Blood samples were collected for: anti-TTG immunoglobulin subclass A (IgA) using enzyme linked immune-sorbent assay (ELISA) and endomyseal antibody (EMA) IgA subclass using indirect immunofluorescence assay, total IgA, CBC, Iron profile, glycosylated hemoglobin (HbA1C), calcium, phosphorus, albumin.

The criterion for selection of patients undergoing intestinal biopsy was any of the following:

I. Positive anti-TTG and positive EMA

II. Positive EMA alone

III. Positive anti-TTG > 50 U/ml alone

IV. Persistently positive anti-TTG at low titer 20 – 50 U/ml (two readings in 6 months)

Following endoscopy, if indicated, 6 duodenal biopsies were obtained including one from duodenal cap. Biopsies were immersed in formalin solution and examined histologically at the Department of Pathology. Formalin-fixed biopsies were stained with hematoxylin and eosin and examined under light microscopy. Biopsies were reviewed by a single pathologist and reported according to Marsh classification [17,18]. The pathologist was blinded to clinical and endoscopic data and serologic results.

### Methods of Serology tests

1) ***Anti -TTG IgA testing*** was undertaken with a commercially obtained ELISA kit (Inova Diagnostics, San Diego, California, USA). In brief, stored serum samples were thawed and diluted with horseradish peroxidase diluent and tested in duplicate at room temperature along with appropriate negative and positive controls. The optical density of each pair of duplicates was converted to an ELISA standard by reference to positive controls. An ELISA cutoff of less than 20 was considered normal and greater than 20, positive. Children with low anti-TTG titer (20–50 U/ml) had a repeat of the test after 6 months. Anti-TTG value < 20 U/ml on the second test defines transient positivity of Anti-TTG and deems intestinal biopsy unnecessary. Persistent positivity of anti-TTG at low titers was considered an indication for intestinal biopsy.

2) ***EMA (IgA)*** in serum was measured using indirect immunofluorescence assay and cryostat sections of monkey esophagus (INOVA Diagnostics Inc., San Diego, California, USA). Serum samples were incubated with substrate for 30 min in moist chamber; sections were then washed with phosphate-buffered saline and incubated for 30 min with fluorescein isothiocyanate. Finally, after washing and applying the mounting medium, sections were examined using fluorescence microscope and the results were reported by comparing with positive and negative controls which were included in every assay. The assays were performed at 3 screening dilutions of 1:5, 1:10, and 1:20. The test result was considered positive when there was a reticulated honeycomb staining of the connective tissue that surrounded the bundles of esophageal smooth muscle.

3) ***Total IgA*:** Serum level of IgA had been assayed using a nephelometric method with the aid of a BN II nephelometer (Siemens, Germany).

The study was approved by the local research and ethics committee of Children’s hospital at King Saud Medical City and had been performed in accordance with the ethical standards laid down in the 1964 Declaration of Helsinki.

### Statistical analysis

The data were analyzed using SPSS pc+ version 16.0 statistical software. Descriptive statistics (mean, standard deviation and proportions) were used to summarize the study variables. Student’s t-test for independent samples was used to compare the mean values of continuous study variables. The 95% confidence intervals for difference of mean were used. Chi-square test and Fisher’s exact tests were used to observe an association between the qualitative study and outcome variables. Sensitivity and specificity values were calculated to evaluate the test procedures (EMA & anti-TTG) in comparison with gold standard (Biopsy). Receiver operated characteristics (ROC) curve was used to determine the best cut-off anti-TTG value with best sensitivity and specificity to diagnose CD. A p value of less than 0.05 was considered statistically significant.

## Results

Of the 106 children screened, 62 were females; age ranged between 8 months and 15.5 years (Mean 8.5 years ± 2.8 years). Mean age at diagnosis of T1D was 6.3 ± 2.9 years (range 0.85 – 11 years). Mean time of duration of T1D was 2.2 ± 2.1 years (range 0 – 8 years).

### Serologic screening

**Figure 1:**
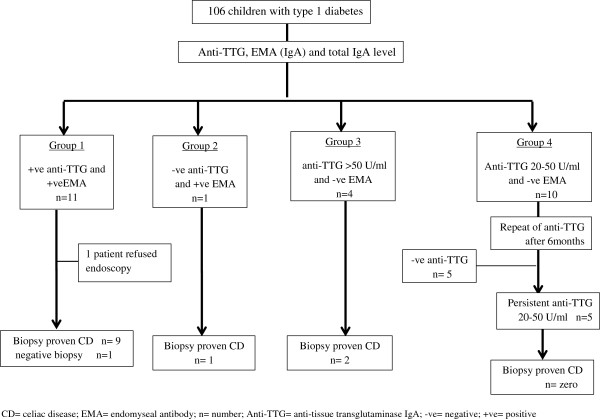
Flowchart for results of the serologic tests and biopsy proven celiac disease.

Of 106 children with T1D, 26 (24.5%) were positive for anti-TTG and / or EMA (Figure 1). In group 4, five of 10 patients with positive low anti-TTG titer (20–50 U/ml) had a negative anti-TTG on a repeated test done after 6 months (Table 1), resulting in overall seroprevalence of 20% (21/106). None of the 10 patients in group 4 had gastrointestinal symptoms. A total of 21 upper endoscopies and duodenal biopsies were performed with 12 patients showing histological features consistent with celiac disease (11.3%) (Figure 1). One patient in group 1 refused endoscopy; he had positive anti-TTG 212 U/ml and positive EMA. The only patient that was EMA and anti-TTG positive but had a normal biopsy had a low positive anti-TTG level (29 U/ml) compared with the other 10 patients with proven atrophic mucosa. Total serum IgA level was in the normal range in all patients. The specificity, sensitivity, positive predictive value, and negative predictive value of the anti-TTG and EMA tests are shown in Table 2.

**Table 1:** Results of repeated anti-TTG test in the group with positive low anti-TTG titer

**Patient**	**First anti-TTG (U/ml)**	**Second anti-TTG (after 6 months) (U/ml)**
**1**	36	67.8
**2**	23	32
**3**	45	62.7
**4**	42	47
**5**	28	33.5
**6**	39	19
**7**	31	17
**8**	29	15
**9**	29.6	16.5
**10**	27	17.5

Anti-TTG= anti-tissue transglutaminase IgA.

**Table 2:** Sensitivity and specificity of anti-TTG and EMA for diagnosis of celiac disease in T1D

	**ANTI-TTG**	**EMA**
**Sensitivity**	91.6%	83.3%
**Specificity**	93.6%	98.9%
**Positive predictive value**	64.7%	90.9%
**Negative predictive value**	98.8%	96.8%

T1D= Type 1 diabetes ; Anti-TTG= anti-tissue transglutaminase IgA; EMA= ednomyseal antibodies.

### Intestinal biopsy proven celiac disease

Clinical, serologic, and histopathological data of 12 children with biopsy proven celiac disease are given in Table 3. Three of 12 patients were diagnosed with CD at time of diagnosis of T1D (25%). Five patients (41.6%) had gastrointestinal symptoms. Gluten free diet has been initiated in all 12 children. Table 4 shows comparison of T1D children with and without celiac disease. Two findings were statistically significant among celiac group: predominance of female gender (92% versus 54%, P = 0.014) and iron deficiency (8.5 μmol/L versus 12.5 μmol/L, P= 0.014).

**Table 3:** Clinical and histopathological profile of 12 children with T1D and celiac disease

**Patient**	**Age(year)**	**Sex**	**Age of onset of T1D(year)**	**Age at diagnosis of CD (year)**	**anti-TTG(<20U/ml)**	**EMA**	**Modified Marsh class**	**Gastrointestinal symptom**
**1**	7.5	M	7	7	117	+ve	3-a	Diarrhea Abdominal pain
**2**	9.5	F	3.5	9.5	212	+ve	3-c	-
**3**	12	F	9	12	283	+ve	3-b	Abdominal pain
**4**	11	F	10	11	236	+ve	3-b	-
**5**	10	F	3	10	103	+ve	3-c	-
**6**	4	F	4	4	109	+ve	1	-
**7**	9.5	F	5	9.5	19	+ve	3-a	-
**8**	11	F	11	11	271	+ve	3-c	Constipation
								Distension
								bloating
**9**	13	F	7	13	151	+ve	3-b	-
**10**	9	F	7	9	287	+ve	3-c	bloating
**11**	8	F	7	8	211	-ve	3-c	Abdominal pain
**12**	6.5	F	5.5	6.5	65	-ve	3-a	-

T1DM= Type 1 diabetes; Anti-TTG= anti-tissue transglutaminase IgA; EMA= ednomyseal antibodies.

**Table 4:** Comparison of T1D children with and without celiac disease

**Study variables mean (± SD)**	**Celiac disease**		**p-value**
	**Yes (12)**	**No (94)**	
Age (year)	9.2 (2.5)	8.5(2.9)	0.38
Age at Diagnosis of T1DM (year)	6.6(2.5)	6.3(3.0)	0.80
Female gender (%)	11(92%)	51 (54%)	0.014*
Duration of T1DM (year)	2.7(2.5)	2.1(2.1)	0.38
Weight(Z-score)	0.04(0.5)	−0.005(1.0)	0.88
Height (Z-score)	0.18(0.56)	−0.02(1.0)	0.50
HbA1c	11.02(2.3)	10.7(2.5)	0.66
Hemoglobin (gm/dl)	12.5(1.0)	13.1(1.0)	0.08
BMI (%)	16.9(2.5)	16.5(3.6)	0.76
MCV (fl)	75.9(6.9)	79.1(5.1)	0.05
MCH (pg)	25.2(2.6)	26.4(2.0)	0.05
Serum iron (normal 9–30.4 μmol/L)	8.5 (3.7)	12.5(5.4)	0.014*
Serum calcium (normal 2.2 –2.7 mmol/L)	2.3(0.07)	2.3(0.16)	0.61
**Autoimmune disease**			
Yes (%)	2 (16.7)	8(8.5)	0.31

T1DM= Type 1 diabetes; HbA1c= glycoselated hemoglobin; *= statistically significant result, -ve= negative; +ve= positive.

### Receiver operated characteristics curve analysis of anti-TTG values

**Figure 2:**
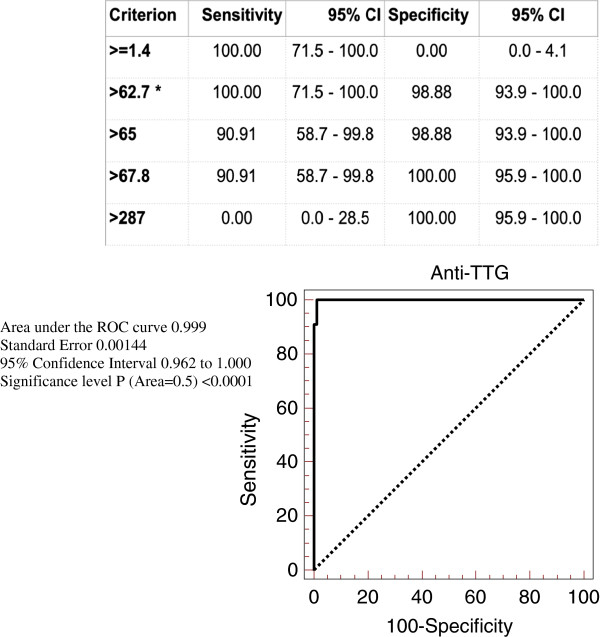
Receiver operated characteristics curve analysis of anti-TTG values.

In order to establish the optimal cut-off / threshold values of anti-TTG for best sensitivity and specificity for diagnosis of CD, we have performed ROC curve analysis by plotting sensitivity against 100-specificity at different cut-off values of anti-TTG (Figure 2). Anti-TTG value at 62.7 U/ml had 100% sensitivity and 98.8% specificity for prediction of diagnosis of CD, with area under the ROC curve of 0.999 and 95% confidence interval of 0.962 to 1 and a significance level of p<0.0001(Area=0.5).

## Discussion

The two most important findings of our study are the high prevalence of CD among Saudi children with T1D (11.3%) and the identification of the lowest positive anti-TTG value (63 U/ml) that best predicts CD in diabetic children with a sensitivity of 100% and specificity of 98.8%. Worldwide, prevalence of CD in children with T1D ranges from 1% to 10% [1-5]. Exceptions being Algeria and Argentina where prevalence rates were reported as 16.4% [19] and 13.9% [20] respectively. Our study identified CD by using rigorous uniform diagnostic criteria including combination of two highly sensitive and specific tests (anti-TTG and EMA), and by performing 6 duodenal biopsies in suspected cases in order to minimize the chance of missing a case of CD that can occur because of the recognized patchiness of histopathological changes. Very Few studies exist in which screening for CD-associated antibodies includes combination of anti-TTG and EMA [2]. Prevalence of CD in our study could have been even higher (12.2%) if we had obtained consent for small bowel biopsy in one child with combined anti-TTG and EMA positive tests. To the best of our knowledge the prevalence rate in our study group is the highest reported in the Middle East and Asia. Such a high prevalence could be related to genetic and environmental factors predisposing to development of CD in Saudi population.

Studies on impact of CD on diabetes control and growth in children with T1D have shown conflicting results [21-23]. In the present study, children with CD had diabetic control and growth parameters equivalent to children without CD. The lack of impact of CD on diabetic control and growth in our study could be related to the relative short duration of diabetes (2.7 years ± 2.5) and that 4 patients were diagnosed with CD at time of diagnosis of diabetes. However, our cross-sectional data do not provide accurate time of onset of CD. While the majority of our patients with CD had no gastrointestinal symptoms, iron deficiency, an index of malabsorption, was prominent in the CD group, a finding that has been shown in another study [24]. The potential for early reversal of abnormalities in indices of intestinal malabsorption (iron and calcium deficiencies) is one of the advantages for screening asymptomatic children for early detection of CD in T1D patients. The predominance of female gender among CD group in the present study has been observed in few studies [25,26], while other studies in different races have shown male predominance [27,28], which likely represents variability of genetic and environmental factors among different races.

Several studies have shown that there is a positive correlation between level of anti-TTG antibodies and degree of villous atrophy with high levels of anti-TTG (> 10 times the upper limit of normal in concentration-dependent antibody tests based on calibration curves) which predict villous atrophy better than low values [11-13]. Patients with positive anti-TTG and normal intestinal biopsy in our study had a low positive anti-TTG level (20–67.8 U/ml) compared with the other 11 patients with higher anti-TTG and proven atrophic mucosa. The low sensitivity and specificity of low positive anti-TTG values for diagnosis of CD have also been observed in other studies [14,15,27]. Another finding that has also been observed by others [16], is that children with T1D, like other autoimmune diseases at risk of CD, could have transiently positive anti-TTG results at low values. Thus children with T1D and low anti-TTG values might be un-necessarily subjected to anesthesia and endoscopy. Transient positivity of CD-specific antibodies in patients with T1D has also been reported with anti-gliadin antibodies [29,30] but was not reported with EMA [31].

By performing ROC curve analysis, we identified anti-TTG value at 62.7 U/ml 3 times the upper limit of normal to have 100% sensitivity and 99% specificity for prediction of diagnosis of CD. Our result support the recommendations by CD working group in European Society of Pediatric Gastroenterology, Hepatology, and Nutrition (ESPGHAN) enclosed in the recent guidelines for the diagnosis of coeliac disease in children and adolescents [32]. In asymptomatic children with CD-associated condition (like T1D), the guidelines recommend performance of upper endoscopy and intestinal biopsies if anti-TTG titres exceeds three times the upper limit of normal. If anti-TTG titres are low positive, that is less than three times upper limit of normal, the guidelines recommend one of two approaches to avoid unnecessary biopsies: first, the child may be followed on a normal gluten containing diet and anti-TTG testing to be repeated in 3 – 6 monthly intervals; the second option involves doing EMA and if positive the child should be referred for intestinal biopsies [32].

## Conclusion

The prevalence rate of CD among Saudi children with T1D is among the highest in the world. Anti-TTG titres more than 3 times the upper limit of normal has very high sensitivity and specificity for diagnosis of CD in T1D children. In order to support the latter finding, larger prospective studies are needed.

## Abbreviations

Anti-TTG: Anti- tissue transglutaminase antibodies IgA; CD: Celiac disease; ELISA: Enzyme linked immune-sorbent assay; EMA: Ednomyseal antibodies; HbA1C: Glycosylated hemoglobin; IgA: Immunoglobulin subclass A; ROC: Receiver operated characteristics; T1D: Type 1 diabetes.

## Competing interests

The authors no conflict of interest to declare.

## Authors’ contributions

All authors read and approved the final manuscript. **ARH:** Is the principal investigator that designed, conducted the study and interpreted the data, and wrote the first draft of this manuscript. **NS**: Provided services associated with the design, conduct of the study, the interpretation of the data, and assisted in writing this manuscript. **MZ**: Collated and assisted in the interpreted the data, and assisted in writing the manuscript. **AA**: Collated and assisted in the interpreted the data, and assisted in writing the manuscript. **IH**: Is the pathologist that was involved in reviewing the intestinal biopsies and assisted in writing and reviewing the manuscript.

## Pre-publication history

The pre-publication history for this paper can be accessed here:

http://www.biomedcentral.com/1471-230X/12/180/prepub
